# High frequency ultrasound of skin involvement in systemic sclerosis – a follow-up study

**DOI:** 10.1186/s13075-015-0853-5

**Published:** 2015-11-19

**Authors:** Roger Hesselstrand, Johanna Carlestam, Marie Wildt, Gunnel Sandqvist, Kristofer Andréasson

**Affiliations:** Department of Clinical Sciences, Section of Rheumatology, Lund University, Lund, Sweden

**Keywords:** Systemic sclerosis, Ultrasound, Skin

## Abstract

**Introduction:**

High-frequency ultrasound offers a potential for objective and quantitative assessment of skin thickness and skin echogenicity in systemic sclerosis (SSc). Few studies have however assessed the longitudinal changes of skin involvement using ultrasound. The aim of the study was to investigate changes in skin thickness in early SSc using high frequency ultrasound during one year of follow-up in comparison to other measurements of skin fibrosis.

**Methods:**

This retrospective study comprised 75 consecutive patients with disease duration shorter than 3 years, in whom ultrasound examination of skin thickness was performed at baseline and at the one year follow-up at five predefined sites.

**Results:**

Repeated ultrasound examination identified significant changes in a majority of patients. In 21 patients, the total sum of skin thickness (TST) increased, while TST decreased in 37 patients. On a group level there were significant decreases in skin thickness of the chest (p = 0.024) and in the TST (p = 0.011) during the observation time. Both baseline and follow-up TST correlated to serum-COMP (r_S_: 0.41; p = 0.001; r_S_: 0.49; p < 0.001), modified Rodnan skin score (mRSS; r_S_: 0.48; p < 0.001; r_S_: 0.48; p < 0.001) and hand mobility in scleroderma (HAMIS; r_S_: 0.30; p = 0.043; r_S_: 0.64; p < 0.001). Changes in TST correlated with changes in serum-COMP (r_S_: 0.30; p = 0.034), changes in mRSS (r_S_: 0.43; p < 0.001) and changes in HAMIS (r_S_: 0.53; p = 0.001) during follow-up.

**Conclusions:**

In early SSc, skin thickness measured by high frequency ultrasound develops in parallel with serum-COMP, mRSS and the HAMIS test. Ultrasound examination of the skin allows for objective assessment of one facet of the complex process of skin fibrosis in early SSc.

## Introduction

Systemic sclerosis (SSc, scleroderma) is characterised by fibrosis, progressive vascular obliteration and the production of autoantibodies. The fibrotic process can target the skin and internal organs and there have been several attempts to quantify both the severity and the activity of such process. The established method of skin assessment is the semi-quantitative scoring (Rodnan skin score, RSS), introduced by Rodnan in 1979, which is used world-wide with variations in definitions of the score grades per anatomical site or number of sites to score [1]. The activity of the fibrosis may also be assessed by the measurement of biomarkers in serum, such as cartilage oligomeric matrix protein (COMP), an extra-cellular matrix molecule produced by fibroblasts during development of fibrosis [2]. The consequences of the fibrosis may be assessed by the hand mobility in scleroderma (HAMIS) test which was specifically developed for patients with SSc [3].

High-frequency ultrasound offers a potential for objective and quantitative assessment of skin thickness and skin echogenicity in SSc. It has been shown to be a valid measurement of skin thickening with a high correlation to RSS [4]. Cross sectional studies have shown excellent inter- and intraobserver variability for dermal thickness in particular with intraclass correlation (ICC) often > 0.8 for interobserver variability and > 0.9 for intraobserver variability [5–7]. In SSc patients with short disease duration, high frequency ultrasound can identify the oedematous phase, which may precede palpable skin involvement, and may thus be useful to identify patients with diffuse skin involvement very early in the disease process [4, 7]. Ultrasound measurements also reflect the severity of the overall fibrotic skin involvement [4]. In addition, dermal finger thickness importantly is associated with nailfold microangiopathy severity assessed by capillaroscopy [8].

Few studies have assessed the longitudinal changes of skin involvement using ultrasound. In a longitudinal pilot study of 16 patients with early SSc and 16 controls, diffuse cutaneous SSc (dcSSc) patients but not limited cutaneous SSc (lcSSc) patients had thicker skin and lower skin echogenicity than controls. Unlike healthy individuals, these SSc patients displayed large intraindividual variation over time; during the follow-up, skin thickness decreased and skin echogenicity increased [6]. In another small study we demonstrated larger in vitro production of proteoglycans from skin fibroblasts from patients with significant dynamics in skin echogenicity [9].

The aim of the present study was to study changes in skin thickness in early SSc using high frequency ultrasound during one year of follow-up in comparison to other measurements of skin fibrosis.

## Methods

### Subjects

All 75 patients fulfilled the 2013 ACR/EULAR criteria [10] and 72/75 fulfilled the 1980 ACR criteria for SSc [11]. Ultrasound examination is performed in all SSc patients in our department at the first visit. For the purpose of this study, patients with short disease duration were selected for repeated ultrasound examination at one-year follow up. This retrospective study comprises 75 patients with disease duration < 3 years at their first ultrasound examination with one year follow up who were all investigated by the same co-author (MW), at the Department of Rheumatology, Skåne University Hospital, Lund, Sweden. All patients had been examined according to the mRSS by one of four physicians. The disease was classified as dcSSc or lcSSc according to the extent of skin involvement [12]. Skin involvement was determined by the modified RSS (mRSS) with palpation of 17 anatomical sites (face, fingers, hands, forearms, upper arms, chest, abdomen, thighs, legs, and feet) and scoring on a 0–3 scale, where 0 = normal skin, 1 = slight thickening, 2 = moderate thickening, and 3 = hidebound skin sclerosis. The scores for all sites were summed to give a total score, with a maximum possible score of 51 [13]. The disease onset was defined as the first non-Raynaud’s manifestation. The mRSS was assessed by one of four physicians in the scleroderma group trained to be concordant in the skin scoring technique. Based on a cohort of 21 patients, the interobserver variability of the mRSS is limited within this group (ICC: 0.943, 95 % CI 0.868-0.976).

### Ethics

The study was approved by the Lund University Ethics Committee, Lund Sweden. All patients gave their written permission according to the Declaration of Helsinki.

### Ultrasound

Skin thickness and skin echogenicity were measured with a high frequency ultrasound (US) scanner (Dermascan, Cortex Technology, Hadsund, Denmark), with 20 MHz transducer. Two scans were obtained of the tissue: a one dimensional A mode image with different echoes defining the interfaces between epidermis, dermis and subcutis, and a two dimensional B mode image with different colours reflecting the differing echogenicities of the skin. The echogenicity in the dermal region was represented on an arbitrary scale (0–255 pixels) where a low value represents high water content, suggesting oedema. In contrast, a high value represents dense extra-cellular matrix content, often seen after the oedema has disappeared. The echogenicity was calculated by outlining a block of skin in which the mean echogenicity was estimated for a selected region [6].

All measurements were made by the same observer (MW) who performed and read the ultrasound examinations, using the same equipment. Five anatomical sites, selected in order to monitor disease development over time were analysed – the dorsal aspect of the mid portion of the proximal phalanx of the right second finger (phalanx), the area (valley) between the metacarpophalangeal joints II and III of the right hand (hand), the dorsal aspect of the right forearm 3 cm proximal of the wrist (forearm), the lateral aspect of the right leg 12 cm proximal of the ankle joint (leg), and the sternum 2 cm distal from the upper part of the manubrium (chest).

The intraobserver variability of this method has been assessed [6]. For the individual patient differences in skin thickness exceeding mean variability two-fold were considered as significant changes.

### HAMIS and COMP

The HAMIS test focuses on the mobility of the fingers and wrist, and comprises nine items, employing differently sized grips and different movements. It reflects the consequences of skin involvement on hand function and was specifically developed for patients with SSc [3].

Serum-COMP was measured with a commercial sandwich enzyme-linked immunosorbent assay (ELISA) using two monoclonal antibodies directed against separate antigenic determinants on the human COMP molecule (AnaMar, Lund, Sweden). The detection limit was 0.1 U/L and the intra- and interassay coefficient of variation was < 5 %.

### Study design

All patients were examined by mRSS and by ultrasound to determine skin thickness and skin echogenicity. Data on serum-COMP and HAMIS were used if measured during the same week as the ultrasound examination. The ultrasound examination was performed at the patient’s first visit to the department during the time period of 1 April 1996 to 1 June 2012. Follow-up data until 1 June 2013 were retrieved. Complete data on ultrasound and mRSS were available for all patients at baseline and at one-year follow-up. Due to the retrospective design serum-COMP and HAMIS data were available in 62 and 54 out of 75 patients at baseline and in 55 and 46 out of 75 patients at follow-up.

### Statistical methods

The distribution of several of the analysed variables was moderately skewed. Non-parametric statistics are used throughout the manuscript. Differences between groups were analysed using the related-samples Wilcoxon Signed Rank test and the Mann–Whitney *U* test. Associations between variables were analysed using the Spearman’s correlation. Results are presented as median (interquartile range (IQR)).

## Results

### Patients

Clinical features of the patients are displayed in Table 1.

**Table 1:** Clinical features at the first ultrasound examination of the skin of 75 SSc patients

Features	Median (IQR)
Age (years)	52.4 (44.8–62.0)
Disease duration (months)	10.9 (7.9–19.1)
mRSS	10.5 (4.0–19)
Serum-COMP (U/L)	12 (10–17) (n = 62)
HAMIS	6.0 (3.5–14) (n = 54)
	n (%)
Women	62 (83)
lcSSc	42 (56)
Immunosuppressive treatment (yes/no)^a^	*39/36 (52/48)*

*mRSS* modified Rodnan skin score, *COMP* cartilage oligomeric matrix protein, *HAMIS* hand mobility in scleroderma test, *lcSSc* limited cutaneous systemic sclerosis, *dcSSc* diffuse cutaneous systemic sclerosis

^a^Cyclophosphamide (n = 16); Azathioprine (n = 10); Methotrexate (n = 7); Mycophenylate mofetil (n = 12). Six patients received two different treatments during the first year of follow-up

At follow-up median mRSS (IQR) changed from 10 (4.0-19) to 9.0 (4.0-21) points, serum-COMP from 12 (10–17) to 12 (8.6-16) U/Land HAMIS from 6.0 (3.5-14) to 7.0 (2.0-16).

### Change in skin thickness and skin echogenicity during the first year of observation

The second ultrasound examination was performed at median (IQR) 14.3 (13.8-15.0) months after the first. Total sum of skin thickness (TST) at baseline and total sum of skin echogenicity (TSE) were inversely correlated (r_S_: −0.51, p < 0.001). Baseline skin thickness and skin echogenicity correlated inversely to changes in the same parameters (r_S_: −0.36, p < 0.001; r_S_: −0.48, p < 0.001 respectively), i.e. the higher the baseline skin thickness the larger was the subsequent reduction in skin thickness. Also, change in TST correlated with change in TSE (r_S_: −0.33, p = 0.003). At the group level there were significant changes (i.e. a reduction) in skin thickness of the chest and the TST during the observation time (Table 2).

**Table 2:** Comparison between skin ultrasound examination at baseline and at the one-year follow-up

Measurement	normal values^a^	baseline	follow-up	change	p
	(95 % CI)	median (IQR)	median (IQR)	median (IQR)	
Skin thickness		(mm)	(mm)	(MM)	
Phalanx	(1.15–2.11)	2.14 (1.92–2.37)	2.05 (1.88–2.33)	−0.02 (−0.22 – 0.13)	0.28
Hand	(0.98–1.47)	1.51 (1.39–1.71)	1.50 (1.34–1.66)	−0.040 (−0.18 – 0.12)	0.14
Forearm	(1.05–1.75)	1.61 (1.44–1.76)	1.55 (1.30–1.76)	−0.080 (−0.23 – 0.10)	0.052
Leg	(1.02–1.64)	1.47 (1.33–1.62)	1.43 (1.29–1.55)	−0.040 (−0.16 – 0.06)	0.060
Chest	(1.04–1.89)	1.63 (1.50–1.92)	1.58 (1.44–1.79)	−0.12 (−0.30 – 0.15)	0.024
TST	(5.62–8.59)	8.53 (7.94–9.03)	8.28 (7.47–8.94)	−0.22 (−0.79 – 0.30)	0.011
Skin echogenicity			(pixels)		
Phalanx	(15.0–44.0)	25.0 (20.0–30.0)	24.5 (21.0–31.0)	0.0 (−4.0 – 4.0)	0.78
Hand	(19.0–47.0)	29.0 (22.5–34.0)	28.0 (22.0–35.0)	0.0 (−5.0 – 6.0)	0.59
Forearm	(22.0–51.0)	38.0 (33.0–45.5)	37.5 (31.5–46.0)	1.0 (−7.0 – 7.0)	0.49
Leg	(31.0–55.0)	47.0 (42.4–56.0)	47.0 (42.4–56.0)	−0.80 (−6.0 – 9.0)	0.94
Chest	(30.0–54.0)	48.0 (41.5–53.0)	48.0 (41.5–53.5)	0.50 (−6.0 – 6.5)	0.53
TSE	(136.0–240.0)	188 (168–212)	187 (166–213)	−2.5 (−23.5 – 30.0)	0.82

*IQR* interquartile range, *TST* total sum of skin thickness, *TSE* total sum of skin echogenicity

^a^normal values from previous study [6]

Significant changes in skin thickness were noticed in a majority of the patients at all five sites (Table 3). Patients who exhibited a significant reduction of skin thickness on the hand and leg also had a decrease in the mRSS. This was not true for patients with a significant increase in the skin thickness on these sites. Patients who exhibited significant increase in skin thickness on the chest and leg had higher serum-COMP levels at baseline. Patients with increased skin thickness on the forearm had worsening of hand function, while the opposite could be seen in patients with reduction in skin thickness on the forearm. We did not see any correlation between change in finger skin thickness and the studied variables.

**Table 3:** Comparison between patients with significant increase or decrease in skin thickness measured by ultrasound at different sites in relation to other assessments of skin fibrosis

Site of US exam	ΔThickness, direction of change (n)	mRSS, baseline (median)	p	ΔmRSS (median)	p	COMP baseline (median)	p	ΔCOMP (median)	p	HAMIS baseline (median)	p	ΔHAMIS	p
Finger	Increase, (18)	9.5	0.250	1.0	0.095	11.7	0.497	1.0	0.150	4.0	0.106	1.5	0.374
	Decrease, (27)	11.0		−1.0		12.3		−1.0		7.5		−1.0	
Hand	Increase, (21)	10.0	0.231	0.0	0.016	12.0	0.750	0.0	0.203	5.0	0.417	0.0	0.540
	Decrease, (27)	10.0		−3.0		12.0		−1.6		7.5		−1.0	
Forearm	Increase, (18)	11.0	0.991	1.5	0.051	15.0	0.467	0.0	0.089	7.0	0.484	4.0	<0.001
	Decrease, (30)	10.5		−1.0		12.0		−1.0		5.0		−1.0	
Leg	Increase, (18)	9.5	0.423	2.0	0.007	15.0	0.029	−0.5	0.986	5.0	0.583	3.0	0.180
	Decrease, (29)	10.0		−1.0		11.7		−0.5		7.0		0.0	
Chest	Increase, (19)	11	0.435	0.5	0.287	15.0	0.029	−1.0	0.940	7.0	0.796	0.0	0.444
	Decrease, (41)	10		0.0		11.7		0.0		6.0		0.0	
Total	Increase, (21)	11	0.802	1	0.019	12.7	0.363	0.0	0.217	5.5	0.344	5.0	0.002
	Decrease, (37)	10		−2		12.0		−1.6		8.0		−1.5	

Increase in hand skin thickness was associated with absence of improved mRSS. Increase in forearm skin thickness was associated with worsening hand function. Increase in skin thickness on leg and chest was preceded by high levels of the fibrosis biomarker COMP

US ultrasound, *mRSS* modified Rodnan skin score, *COMP* cartilage oligomeric matrix protein, *HAMIS* hand mobility in scleroderma test

Note to reviewer: The numbers describing COMP baseline with Δthickness on leg and chest are by chance identical, this has been controlled

### Change in skin thickness and skin echogenicity in relation to disease duration

We identified a weak inverse correlation between TST and disease duration (r_S_: −0.22, p = 0.056) which was not statistically significant, although indicating trends towards higher skin thickness when disease duration was shorter.

### Correlation between ultrasound measurements and mRSS, serum-COMP and HAMIS

Fibrosis is challenging to evaluate. Considering the simplification that ultrasound mainly measures skin thickness, mRSS measures severity, serum-COMP reflects fibrotic activity and HAMIS the consequences of fibrosis on hand function, all these measures are closely related, yet examine different facets of the disease. Indeed, the ultrasound measurements correlated to serum-COMP, mRSS, and HAMIS both at baseline and the one-year follow-up (Table 4). At baseline, TST correlated with serum-COMP (r_S_: 0.41, p = 0.001), mRSS (r_S_: 0.48, p < 0.001) and HAMIS (r_S_: 0.30, p = 0.043). At follow-up TST correlated with serum-COMP (r_S_: 0.49, p < 0.001), mRSS (r_S_: 0.48, p < 0.001) and the correlation to HAMIS increased further (r_S_: 0.64, p < 0.001). At baseline, TSE correlated with serum-COMP (r_S_: −0.28, p = 0.025), mRSS (r_S_: −0.40, p = 0.001) but the correlation to HAMIS was not significant (r_S_: −0.28, p = 0.066). At follow-up, the correlation between TSE and serum-COMP was no longer significant (r_S_: −0.16, p = 0.23), whereas there was still a weak correlation to mRSS (r_S_: −0.24, p = 0.044) but no significant correlation to HAMIS (r_S_: −0.26, p = 0.087).

**Table 4:** Correlations between skin ultrasound examination and serum-COMP, mRSS, and HAMIS

Baseline	r_S_ (p)		
	serum-COMP	mRSS	HAMIS
TST	0.41 (0.001)	0.48 (<0.001)	0.30 (0.043)
TSE	−0.28 (0.025)	−0.40 (0.001)	−0.28 (0.066)
1-year follow-up		r_S_ (p)	
	serum-COMP	mRSS	HAMIS
TST	0.49 (<0.001)	0.48 (<0.001)	0.64 (<0.001)
TSE	−0.16 (0.23)	−0.24 (0.044)	−0.26 (0.087)
		r_S_ (p)	
	serum-COMP change	mRSS change	HAMIS change
Change in TST	0.30 (0.034)	0.43 (<0.001)	0.53 (0.001)

*COMP* cartilage oligomeric matrix protein, *mRSS* modified Rodnan skin score, *HAMIS* hand mobility in scleroderma test, *TST* total sum of skin thickness, *TSE* total sum of skin echogenicity

On average, changes in TST correlated with changes in serum-COMP (r_S_: 0.30, p = 0.034), mRSS (r_S_: 0.43, p < 0.001) and strongly with changes in HAMIS (r_S_: 0.53, p = 0.001) during the same time period, i.e. a reduction in skin thickness is accompanied by decreasing COMP production, reversal of mRSS and improved hand function (Table 4).

## Discussion

The skin involvement in SSc is a complex process comprising ischaemia, inflammation and fibrosis and it is difficult to distinguish between thick, hard or hidebound skin. High-frequency ultrasound offers a potential for quantitative assessment of skin thickness and skin echogenicity in SSc. In this longitudinal study during one year of follow-up we identified significant changes in a majority of patients on an individual level. Skin thickness decreased and skin echogenicity increased at all of the five predefined areas investigated. While we noticed a reduction of skin thickness on the group level, there was a great individual variation with several patients showing worsening of skin thickness during the study period. Both the decreased thickness and increased echogenicity were expected and in line with results of our previous smaller study where most patients gradually became more similar to the control population during follow-up [6]. This is further exemplified by the inverse correlation between skin thickness and disease duration, which strengthens the notion that in SSc, the skin is thick in early disease whereas the abnormality of the skin in long-term disease is mostly related to the skin being hard and hidebound. This may be exemplified by the correlation between mRSS and TST, which was 0.48 at baseline in this study with patients with disease duration of 1.10 years. In a previous study of patients with disease duration of two years or less the correlation between mRSS and TST was 0.66 [4]. However, when those patients were subdivided the correlation was 0.81 for patients with disease duration less than one year but only 0.19 for patients with disease duration of one to two years. Similar, but less pronounced, differences were seen in the present study where we found a correlation of 0.63 in patients with disease duration shorter than one year and 0.40 in patients with disease duration of one to three years. Our interpretation is again that mRSS reflect the increased skin thickness mainly in early disease and that skin thickness and the mRSS can be disconnected later on, most notably on the fingers and back of hands. One year of follow-up was enough to detect significant decrease or increase in ultrasound examination of skin thickness in a majority of patients although a majority of our patients were not yet normalised in skin thickness at this time-point. Of interest, serum-COMP was associated with dynamics of fibrosis on the leg and chest which might reflect the substantial biomass of the skin from these sites compared to the finger and hands. HAMIS, on the contrary, was associated with change in skin thickness on the forearm which may indicate novel speculation on the biological correlates on reduced hand function in SSc. Finally, skin thickness of the hand and lower leg was associated with change in mRSS. This finding might remind us that skin changes in the distal parts of the arms and legs contribute to 30 of the total maximum 51 points in the mRSS.

TST, both at baseline and at follow-up, correlated with serum-COMP, and TSE correlated with serum-COMP at baseline, as previously shown [2], indicating that fibrotic activity and oedema occur simultaneously. However, TST correlated with HAMIS to a higher degree at follow-up than at baseline. It appears that HAMIS specifically measures the consequences of the fibrotic activity in the hands and in earlier stages of disease the skin may be softer although thick [14]. A likely synthesis of the information from ultrasound, serum-COMP and HAMIS measurements is that initially increased skin thickness and fibrotic activity may be accompanied by swollen but soft skin, thus less correlated to HAMIS. In advanced disease there is less oedema and the increased skin thickness may be caused by the accumulation of matrix components leading to fibrosis and impaired hand mobility. SSc is a heterogeneous disease with profound interindividual variations in dynamics in fibrotic development. For the time being we are cautious to suggest a certain clinically relevant change in ultrasound measurements for an individual patient, not at least since a normalisation of TST or TSE still can be followed by an impaired function.

Strengths of this study are the use of one single observer for all ultrasound examinations and a low number (four) of observers performing mRSS. Limitations of the study are the retrospective design resulting in incomplete data on serum-COMP and HAMIS-measurements and the small sample size making subgroup analyses uncertain. Also, although the correlations observed are statistically significant and point in the same direction they are often of weak to moderate strength. Finally, a majority of the patients received immunosuppressants including prednisone between the two ultrasound examinations. It cannot be ruled out that some of the changes identified in this study on both skin thickness and other variables studied are partly explained by these medications.

## Conclusions

Fibrosis is a complex process to analyse objectively. Ultrasound offers a unique perspective to evaluate skin thickness objectively. In early SSc, skin thickness measured by high frequency ultrasound develops in parallel with serum-COMP, mRSS, and the HAMIS test. Ultrasound examination of the skin allows for objective assessment of one facet of the complex process of skin fibrosis in early SSc.

## Abbreviations

ACR/EULAR: American College of Rheumatology/European League against Rheumatism
COMP: Cartilage oligomeric matrix protein
dcSSc: Diffuse cutaneous systemic sclerosis
ELISA: Enzyme linked immunosorbent assay
HAMIS: Hand mobility in scleroderma
ICC: Intraclass correlation
IQR: Interquartile range
lcSSc: Limited cutaneous systemic sclerosis
mRSS: Modified Rodnan skin score
RSS: Rodnan skin score
SSc: Systemic sclerosis
TSE: Total sum of skin echogenicity
TST: Total sum of skin thickness
